# Supplementary material for: Correlation between Ferromagnetic Layer Easy Axis and the Tilt Angle of Self Assembled Chiral Molecules
Source: Molecules. 2020 Dec 20;25(24):6036. doi: 10.3390/molecules25246036 (PMC7765850; doi:10.3390/molecules25246036)
Supplement: Supplementary file 1 [file molecules-25-06036-s001.pdf]

Correlation Between Ferromagnetic layer Easy Axis and the Tilt Angle of Self Assembled Chiral Molecules – supporting information

Nir Sukenik[1], Francesco Tassinari[2], Shira Yochelis[1], Oded Millo[3], Lech Tomasz Baczewski[4], Yossi Paltiel[1,*]

[1] Applied Physics Department and the Center for Nanoscience and Nanotechnology, The Hebrew University of Jerusalem, 91904 Jerusalem, Israel
[2] Department of Chemical and Biological Physics, Weizmann Institute of Science, Rehovot, 76100 Israel
[3] Racah Institute of Physics and the Center for Nanoscience and Nanotechnology, The Hebrew University of Jerusalem, 91904 Jerusalem, Israel
[4] Magnetic Heterostructures Laboratory, Institute of Physics, Polish Academy of Sciences, Al. Lotnikow 32/46, 02-668 Warszawa, Poland
* Correspondence: paltiel@mail.huji.ac.il

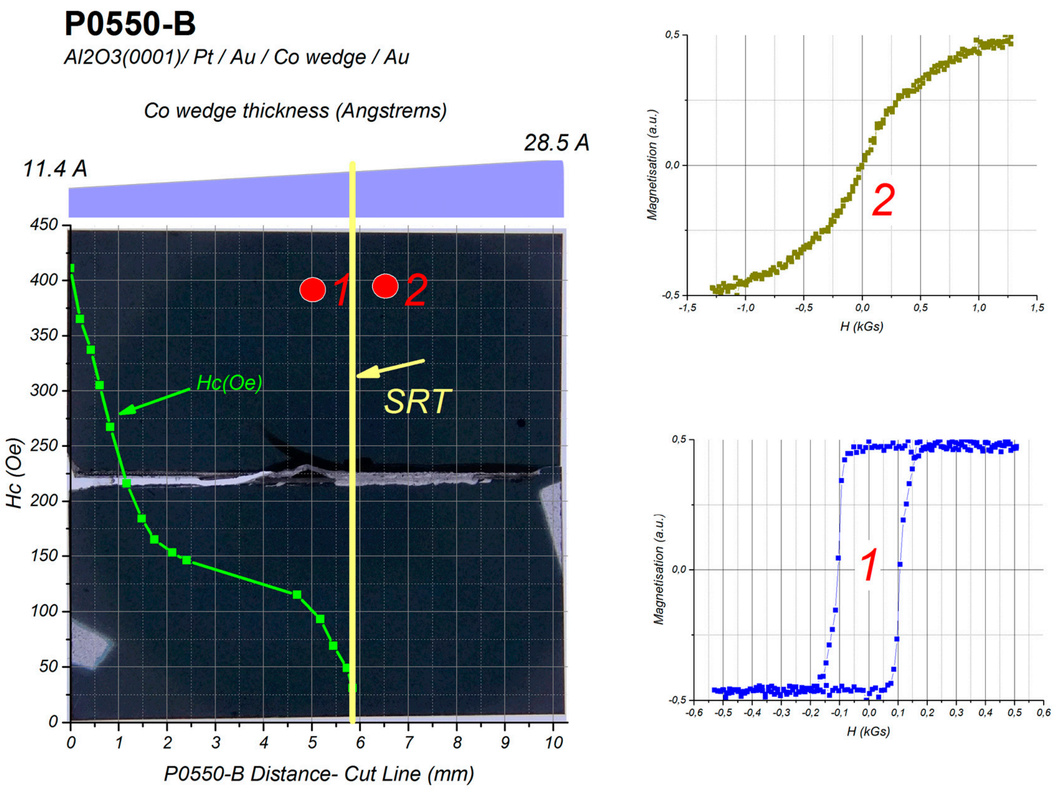

Figure S1- Magnetic characterization of the ferromagnetic substrate with Co wedge layer. Top of the Fig S1 - the thickness of the Co layer as a function of the position on the sample is shown. Left image of the sample - the coercive field measured for the area with OoP easy axis plotted as a green line. Red points 1 and 2 on the sample image correlate with graphs 1 and 2 on the right side on the Fig.S1 showing the out of plane hysteresis loops measured by polar magneto optical Kerr effect (P-MOKE). Yellow line denotes the critical Co thickness where the spin reorientation transition (SRT) occurs and the magnetization direction changes from perpendicular to in-plane.

Figure S1. Shows the details and characterization of the ferromagnetic substrate with Co wedge layer. Magnetization characterization measurements were done using polar magneto optical Kerr effect microscopy.

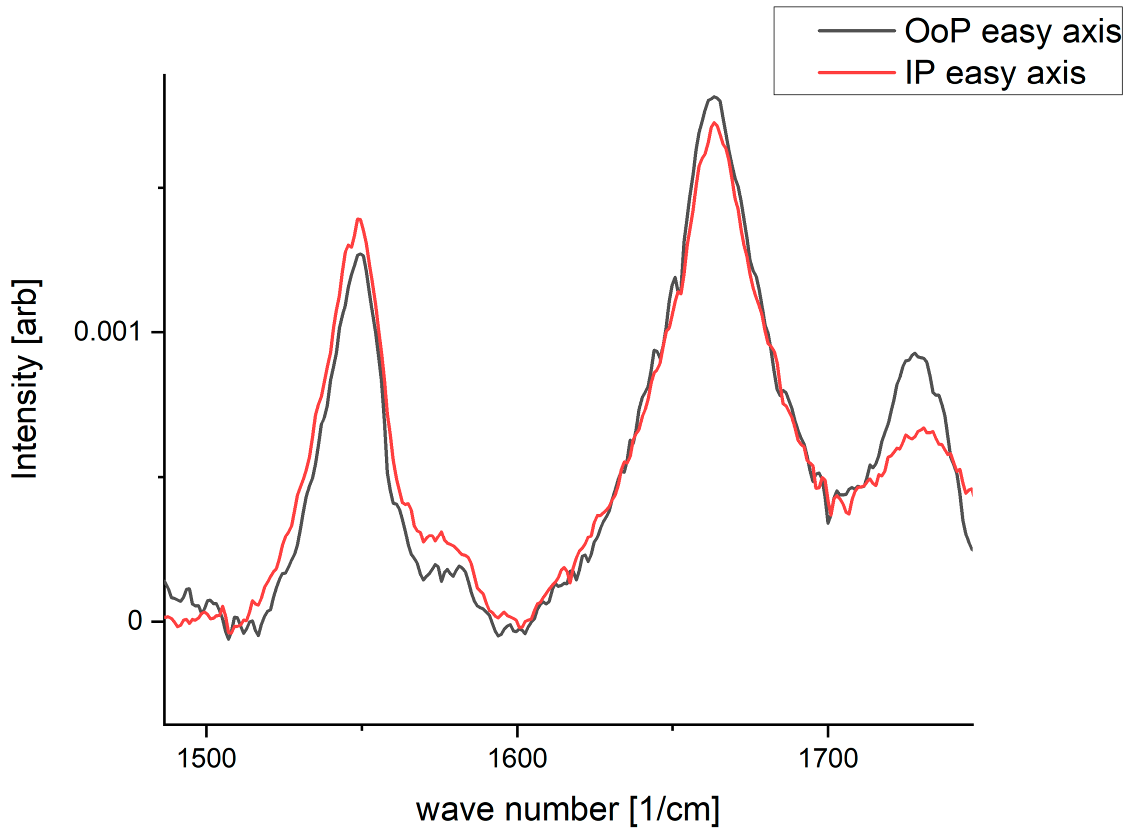

Figure S2- Polarization modulation–infrared reflection–absorption mode in FTIR measurement of the SAM tilt angle for both IP and OoP easy axis. Analysis of the intensity ratio between the peaks as done in ref. 8, gives a tilt angle of about~60° to the sample normal for the IP easy axis and ~ 55° for the OoP easy axis. These are similar differences between IP to OoP as measured using MFM.

Figure S2 shows results of the molecular monolayer bond tilt angle measured by Polarization modulation- infrared reflection absorption mode in FTIR (PM-IRRAS) on both IP easy axis areas of the FM and OoP easy axis areas of the FM. The PM-IRRAS spectra were measured on a Nicolet 6700 FTIR instrument equipped with a PEM-90 photoelastic modulator (Hinds Instruments, Hillsboro, OR) at an 80° angle of incidence. The orientation of the peptide monolayer on the substrate was calculated using the equation

$$\frac{I_1}{I_2} = 1.5 * \left[\frac{(3cos^2\gamma - 1)(3cos^2\theta_1 - 1) + 2}{(3cos^2\gamma - 1)(3cos^2\theta_2 - 1) + 2}\right]$$

where I_1 and I_2 are the intensity of the amide I and amide II bands, θ_1 and θ_2 are the angles between the transition moment of the two bonds and the helical axis (which were found in the literature to be 39° and 75°, respectively) and γ is the tilt angle of the helix in respect to the surface normal.
